# Household health expenditure does not improve people’s subjective well-being in China

**DOI:** 10.3389/fpubh.2024.1402191

**Published:** 2024-09-12

**Authors:** Weiwei Wang, Yan Sun, Gen Li, Yingde Tang

**Affiliations:** ^1^School of Economics and Management, Jiangsu University of Science and Technology, Zhenjiang, China; ^2^College of Humanities and Social Sciences, Jiangsu University of Science and Technology, Zhenjiang, China; ^3^College of Earth and Environmental Sciences, Lanzhou University, Lanzhou, China

**Keywords:** medical expenses, health protection expenses, subjective well-being, Chinese family panel studies, China

## Abstract

**Introduction:**

Household health expenditure plays a crucial role in the daily spending of individuals. Meanwhile, the attention of the public to subjective well-being (SWB) is constantly increasing in China. Household health expenditure could reduce real family income, harming personal SWB. However, the aim of household health expenditure is to improve the physical condition of an individual, and improvements in individual health could enhance personal SWB. Therefore, the effect of household health expenditure on personal SWB is uncertain; hence, it is essential to assess the effects of household health expenditure on the SWB of Chinese residents.

**Methods:**

The Chinese family panel studies database from 2016 to 2020 was applied in this study. A fixed effects model was used to examine the impact of household medical and health protection expenses on personal SWB. Fixed effects instrumental variable regression and propensity score matching were then used to conduct robustness testing.

**Results:**

On the basis of a fixed effects model, it was found that household medical and health protection expenditure did not improve the happiness and life satisfaction of individuals; rather, household health protection expenditure could significantly reduce personal happiness. Fixed effects instrumental variable regression and propensity score matching analysis supported these results. Household health protection expenditure had a greater negative impact on the happiness and life satisfaction of females compared with males.

**Conclusion:**

Household health expenditure does not improve the SWB of individuals in China; this has certain significance for the formulation of relevant policies.

## 1 Introduction

### 1.1 Background

The subjective well-being (SWB) has become a hot topic in academic research. With the tremendous development of the Chinese economy, the attention paid to SWB by the government and general public is also constantly increasing in China, personal SWB has gradually become an important aspect of national sustainable development considerations, “Happiness China” has become a hot topic in current society (1, 2).

Modern mainstream economics is characterized by maximizing utility under constraints, and utility is a weak ordering of commodity bundles (3). Hence, consumption is often considered very important for achieving and maintaining well-being (4). As an aspect, healthcare consumption is aimed at improving personal health, and researcher suggests that happiness is strongly correlated with individual health (5), where the individual health includes subjective health and objective health (6). Research has also shown that physical and mental diseases can cause a significant decline in happiness (7). The theory of the hedonic treadmill suggests that a person can adapt almost completely to most life events; however, many studies have shown that individuals have extreme difficulty in completely adapting to degenerative diseases (5, 8). The effect of health on SWB is also related to age; for those >85 years old, physical health was not found to be an influential factor of SWB (9). Two-way causality should be considered when studying the relationship between health and happiness (10, 11); to address this endogeneity concern, Crivelli and Lucchini (5) used the generalized method of moment (GMM) to demonstrate that health is one of the most important factors affecting happiness.

Healthcare expenditure will increase with increasing income, just like other consumption expenditure (12). Health, like education, is also a form of human capital that can promote income growth (13). Health expenditure is an important part of household consumption expenditure, this expenditure is a part of gross domestic product statistics that can promote economic growth (12). However, this expenditure can also reduce other consumption expenditure, potentially damaging economic development, as well as reducing the real income of a household, harming personal SWB. The aim of household health expenditure is to improve the physical condition of individuals, and improvements in individual health could enhance personal SWB. Therefore, the effects of health expenditure on individual SWB remain uncertain.

This study attempts to answer the following question: what is the impact of household health expenditure on personal SWB in China? Panel data from the China family panel studies (CFPS) database were used to study the effect of household health expenditure on the SWB of individuals. In this study, SWB included happiness and life satisfaction, and household health expenditure included household medical expenses and household health protection expenses dummy. To address the biases caused by individual heterogeneity, individual fixed effects models were applied to estimate this impact, and fixed effects instrumental variable (IV) regression and propensity score matching (PSM) model were used for robustness testing. The main contributions of this paper are as follows: (1) this paper answer the effect of household health expenditure on the SWB of individuals in China, which is interesting for a wide range of stakeholders. (2) This study analyzed the possible main reasons for the effect of household health expenditure on the SWB of individuals in China. (3) This study analyzed the gender differences of household health expenditure on the SWB of individuals.

### 1.2 Literature review

The utility theory in western economics holds that consumption is very important for achieving and maintaining well-being. There have been numerous studies on the effects of consumption on subjective well-being (SWB) of individuals, but the effects are inconsistent for different regions and consumption categories. The using of panel data has shown that consumption can enhance personal happiness in China (14). Evidence from Vietnam suggests that material consumption is not necessarily associated with increased well-being; for those who take better care of their possessions, material consumption can increase well-being (15). In Turkey, only the consumption of durable goods is correlated with life satisfaction (16). Data from the U.S indicate that only leisure consumption of nine specific consumption categories is positively correlated with SWB (17). For different consumption categories, studies indicate that luxury consumption leads to increased SWB (18), hazardous consumption lowers the life satisfaction of students (19), and experiential consumption, rather than materialistic consumption, promotes personal SWB (20). It has been pointed out that the effect of consumption on SWB is related to the motives that drive the acquisition of a product and how people relate to the items they are buying (21). The impact of consumption on well-being is also related to the purpose of consumption, in which a material value orientation of consumption will reduce personal well-being (22).

For researches on the impact of health expenditure on SWB, there are numerous studies about the effects of government health expenditure on residents’ SWB. Most studies suggest that public health expenditure could enhance residents’ SWB (23). For example, Bjørnskov et al. (24) used cross-sectional data of 74 countries to obtain a positive relationship between public health investment and residents’ SWB. Similar conclusions have been drawn from research on Finland and China (23, 25). For researches on the relationship between health expenditure and SWB, there are a lot of researches on the impact of medical insurance on residents’ SWB. A large number of researches showed that urban and rural resident basic medical insurance could significantly improve the SWB of Chinese residents (26, 27). The studies in US also showed that the health insurance have a positive impact on residents’ life satisfaction, the possible reasons are that health insurance reduces uncertainty (28).

However, there are few researches concern the impact of household health expenditure on residents’ SWB so far. Household health expenditure is an important aspect of consumption, this expenditure will improve people’s health and thus enhance their SWB. On the other hand, this expenditure will reduce other consumption and household savings levels, that will reduce people’s SWB. So, it is still uncertain for the impact of household health consumption on residents’ SWB. In this study we will try to fill the gaps, the conclusions will provide assistance to a wide range of stakeholders.

## 2 Materials and methods

### 2.1 Data sources and samples

In this study, CFPS database was used to investigate the impact of household health expenditure on the SWB of individuals. The CFPS is a major social science project implemented by the Institute of Social Science Survey, Peking University. The CFPS aims to reflect changes in Chinese society, economy, population, education, and health by tracking and collecting data at the individual, family, and community levels, providing data for academic research and policy decision-making. The survey started in 2010 and has been conducted every 2 years; data for 2020 have recently been released. The variable happiness was not investigated or survey content was inconsistent with the subsequent investigation for adult before 2014. For the year of 2014, it is lack of individual perceived health status variable. The health status of an individual is related to health expenses and can also affect personal SWB. Since the 1950s, perceived health has been one of the most commonly used health indicators in sociological health research (29). Existing research suggests that self-ratings of health are a valid measure of health status (30), even if they may be modified by age or culture (31). If omitted, this can cause omitted variable bias, which in turn causes inconsistencies in results (32). Therefore, the data selected in this article are from three panel surveys, including those in 2016, 2018, and 2020. By merging personal and household databases of CFPS, removing data that failed to match, there are 11,030 households and 27,673 individuals in 2020, 13,879 households and 36,735 individuals in 2018, 13,867 households and 36,213 individuals in 2016. However, there are missing values for many variables, that will reduce the size of observations in regression analysis.

### 2.2 Variables

A description of the variables in this study is shown in Table 1. The dependent variable of this study was SWB; the most widely accepted concept regarding SWB is that it is a multidimensional concept composed of cognitive and emotional aspects of well-being (9, 33–35). Happiness is generally closer to the emotional aspect of well-being and experienced well-being (36–38). Overall life satisfaction is the cognitive aspect of SWB, and life satisfaction is also known as evaluative well-being (34). In this study, happiness was used as the emotional experience measurement of SWB, and life satisfaction was used as the measurement of evaluative SWB. The CFPS dataset was used to study the SWB of individuals. In this dataset, respondents answered the question “Are you happy?” to measure happiness on an 11-point scale, with 0 being the lowest and 10 the highest. Respondents also answered the question “Are you satisfied with your life?” to measure life satisfaction on a 5-point scale, with 1 being very unsatisfied and 5 very satisfied.

**Table 1:** Variable descriptions in the model.

Variables	Variable descriptions	Mean (SD)/Frequency (%)
Happiness	Score from 0 to 10, “0” be the lowest score	7.549 (2.127)
Life satisfaction	Score from 1 to 5, “1” be the lowest score	3.857 (1.016)
Ln HME	Natural logarithm of medical expenses in the past 12 months	6.626 (2.971)
HPE dummy	Expenses incurred = 1	6,601 (16.632)
	No expenses = 0	33,087 (83.368)
Self-rated health	Excellent	15,076 (15.092)
	Very good	17,657 (17.675)
	Good	37,828 (37.868)
	Fair	13,497 (13.511)
	Poor	15,834 (15.850)
Ln household income	Natural logarithm of household income	10.818 (1.205)
Ln HCE	Natural logarithm of household consumption expenses	10.603 (0.971)
Chronic Diseases	Dummy, had chronic diseases within six months = 1	13,567 (15.992)
	No chronic diseases within six months = 0	71,270 (84.008)
Hospitalized	Dummy, hospitalized in the past year = 1	10,090 (11.296)
	Not hospitalized in the past year = 0	79,235 (88.704)
Demographic variables		
Female	Female = 1	50,432 (50.129)
	Male = 0	50,173 (49.871)
Age	Continuous variable	45.162 (18.801)
Marriage dummy	Having a spouse in marriage = 1	68,493 (76.980)
	Else = 0	20,482 (23.020)
Education dummy	Lower than primary school	30,912 (32.568)
	Primary school	21,718 (22.882)
	Middle school	21,769 (22.935)
	High school	11,467 (12.081)
	University education	8,715 (9.182)
	Postgraduate	333 (0.351)
Others		
Urban resident	Urban = 1	47,444 (49.088)
	Rural = 0	49,206 (50.912)
Year dummy	Dummy of survey year	–
Province dummy	Dummy indicators included 31 provinces, municipalities, and autonomous regions of China and are not described here	–

SD, standard deviation; HME, household medical expenses; HPE, household health protection expenses; HCE, household consumption expenses.

The core explanatory variable in this study was household health care expenditure. This comprised two sub-variables: household medical expenses and household health protection expenses. Household medical expenses comprised direct payments made by the respondent and their family over the past 12 months; these excluded payments that had already been reimbursed and those expected to be reimbursed. Existing research has generally used natural logarithms for large numbers, such as income and consumption, when used econometrically (39, 40); hence, we converted household medical expenses into natural logarithms. Household health protection expenses comprised those related to exercise for fitness and the purchasing of related products and equipment, as well as healthcare products. Most families do not have health protection expenses; only 20% of households had health protection expenses in 2020 according to CFPS, so household health protection expenses were transformed into dummy.

Control variables included demographic variables, individual perceived health status, natural logarithm of household income, natural logarithm of household consumption expenses, urban resident dummy, province dummy, and year dummy. A description of these variables is shown in Table 1. The demographic variables included gender, age, marriage dummy, and education dummy. Individual health condition and household income are related to household health expenditure, and individual health condition and household income also affect personal SWB; hence, individual health condition and household income were added as control variables. The health condition of an individual included perceived health, chronic diseases dummy, and hospitalized dummy. Perceived health, as one subjective evaluation of health status, is a valid measure of health status (31), and is one of the most commonly used health indicators in sociological health research (29). In addition, chronic diseases dummy within 6 months and hospitalized dummy in the past year were used as objective health conditions. Because chronic diseases dummy and hospitalized dummy had very few values of 1 (see Table 1), perceived health was required to measure the health status of individuals.

### 2.3 Model setting

Ordinary least squares do not consider unobservable individual heterogeneity; this individual heterogeneity may be related to the explanatory variable, and individual heterogeneity may also affect the dependent variable, causing inconsistent estimation results. To address this endogeneity issue, a fixed effects model was used to examine the impact of household medical expenses and household health protection expenses on the SWB of individuals. The model is expressed as follows:


(1) $$\begin{array}{c} SWB_{i,t}=\beta_{0}+\beta_{1}\mathrm{LnHM}E_{i,t}+\beta_{2}HPE\;\mathrm{dumm}y_{i,t}\\ +\sum \beta_{k}\mathrm{contro}l_{i,t}+\mu_{i}+\mu_{c}+\lambda_{t}+\varepsilon_{i,t} \end{array}$$

where, *LnHME_i,t_* is the natural logarithm of household medical expenses for individual *i* at time *t*, *HPE dummy_i,t_* is the household health protection expenses dummy for individual *i* at time *t*, *control_i,t_* are control variables for individual *i* at time *t*, *μ_i_* is the individual fixed effect, *μ_c_* is the province fixed effects, *λ_t_* is the time fixed effect, and *ε_i,t_* is the error term.

A fixed effects model can address the omitted variable bias caused by individual heterogeneity that does not change over time (Equation 1). However, there may also be changes in unobservable variables over time and with different individuals, and such variables may affect both household medical expenses and SWB. Because of these endogeneity concerns, fixed-effects IV regression was used to conduct robustness testing. When there is a significant difference between two sets of covariates, regression analysis cannot generally obtain robust estimation results. Propensity score matching can separate a relatively balanced covariate from an observed sample with imbalanced covariates, giving more robust estimated results with less sensitivity to the form of the function (41). Hence, PSM was used to study the impact of household health protection expenses dummy on SWB as a part of the robustness testing.

## 3 Results

### 3.1 Descriptive statistics

The means and standard deviations, or frequencies and percentages, of all variables are shown in Table 1; the correlations between dummy variables and SWB are shown in Table 2; and the correlation matrices of the main continuous variables are shown in Table 3. The Pearson’s correlation coefficient between the natural logarithm of household medical expenses and SWB was significantly negative; the coefficient with happiness was −0.017 (*p* < 0.01), and the coefficient with life satisfaction was −0.019 (*p* < 0.01). However, the Pearson’s correlation coefficient between the natural logarithm of household health protection expenses and SWB was significantly positive, confirmed by the student’s *t-*test (Table 2). The Pearson’s correlation coefficient between household income and SWB was significantly positive. Happiness and life satisfaction increased along with improvements in self-rated health, and the happiness and life satisfaction of female respondents were significantly higher than those of male respondents (Table 2).

**Table 2:** Descriptive statistics of dummy variables in the model.

Variables		Happiness	Life satisfaction
		Mean (SD)/*p-*value	Mean (SD)/*P-*value
HPE dummy	Expenses incurred	7.596 (1.951)	3.870 (0.932)
	No expenses	7.538 (2.161)	3.854 (1.032)
	*P-*value	0.014	0.099
Self-rated health	Excellent	8.290 (2.024)	4.198 (0.959)
	Very good	7.891 (1.898)	3.947 (0.946)
	Good	7.465 (1.967)	3.818 (0.954)
	Fair	7.128 (2.189)	3.714 (1.051)
	Poor	6.838 (2.523)	3.680 (1.174)
	*P-*value	<0.001	<0.001
Female	Female	7.572 (2.130)	3.886 (1.008)
	Male	7.525 (2.123)	3.829 (1.024)
	*P-*value	0.009	<0.001
Marriage dummy	Having a spouse in marriage	7.572 (2.094)	3.894 (1.016)
	Else	7.247 (2.204)	3.729 (1.009)
	*P-*value	<0.001	<0.001
Education dummy	Lower than primary school	7.491 (2.483)	3.897 (1.117)
	Primary school	7.527 (2.251)	3.827 (1.066)
	Middle school	7.551 (2.050)	3.874 (0.970)
	High school	7.582 (1.858)	3.841 (0.891)
	University education	7.641 (1.670)	3.814 (0.839)
	Postgraduate	7.606 (1.597)	3.869 (0.736)
	*P-*value	<0.001	<0.001
Chronic diseases	Had chronic diseases	7.277 (2.291)	3.832 (1.064)
	No chronic diseases	7.539 (2.087)	3.862 (1.007)
	*P-*value	<0.001	0.002
Hospitalized	Hospitalized	7.339 (2.350)	3.876 (1.074)
	Not hospitalized	7.576 (2.093)	3.855 (1.008)
	*P-*value	<0.001	0.051
Urban resident	Urban	7.609 (2.019)	3.850 (0.986)
	Rural	7.494 (2.229)	3.869 (1.046)
	*P-*value	<0.001	0.007

SD, standard deviation; HME, household medical expenses; HPE, household health protection expenses; HCE, household consumption expenses. All significance levels of student’s *t-*test corresponding to *p*-values are two tailed, *F* test corresponding to *P*-values are single tailed.

**Table 3:** The correlation matrix of the main continuous variables.

Variables	(1)	(2)	(3)	(4)	(5)	(6)	(7)
(1) Happiness	1.000						
(2) Life satisfaction	0.462^***^	1.000					
(3) Age	−0.029^***^	0.133^***^	1.000				
(4) In household income	0.084^***^	0.047^***^	−0.149^***^	1.000			
(5) In HCE	0.058^***^	0.012^***^	−0.166^***^	0.558^***^	1.000		
(6) In HPE	0.013^***^	0.009^**^	−0.010^***^	0.243^***^	0.296^***^	1.000	
(7) In HME	−0.017^***^	−0.019^***^	0.074^***^	0.054^***^	0.226^***^	0.074^***^	1.000

SD, standard deviation; HME, household medical expenses; HPE, household health protection expenses; HCE, household consumption expenses. ^*^p < 0.05, ^***^p < 0.01.

### 3.2 Fixed effects model results

The fixed-effects modeling results are shown in Table 4. For models 1 and 2, the dependent variable was happiness; model 1 was a fixed-effects model that did not include self-rated health, while model 2 incorporated self-rated health. The effect of household medical expenses on happiness was not significant in either model. However, a significantly negative impact of household health protection expenses on happiness was observed. In model 2, when keeping other variables constant, the average happiness of an individual with household health protection expenditure was 0.114 lower than that of an individual without such expenditure.

**Table 4:** Individual fixed effect model regression results.

Dependent variable	Model 1	Model 2	Model 3	Model 4
	Happiness	Happiness	Life satisfaction	Life satisfaction
	Fixed effect	Fixed effect	Fixed effect	Fixed effect
Ln HME	−0.004 (0.006)	−0.002 (0.006)	−0.003^*^ (0.002)	−0.002 (0.002)
HPE dummy	−0.109^***^ (0.038)	−0.114^***^ (0.038)	−0.021^*^ (0.013)	−0.024^*^ (0.013)
Ln household income	0.080^***^ (0.023)	0.079^***^ (0.023)	0.021^***^ (0.007)	0.021^***^ (0.007)
Ln HCE	0.020 (0.026)	0.018 (0.026)	0.001 (0.008)	0.001 (0.008)
Age	−0.026 (0.040)	−0.020 (0.041)	−0.014 (0.009)	−0.012 (0.009)
Urban	0.083 (0.080)	0.095 (0.079)	−0.013 (0.025)	−0.010 (0.024)
Female	−0.082 (0.550)	−0.057 (0.552)	0.006 (0.125)	0.001 (0.125)
Marriage dummy	0.352^***^ (0.117)	0.347^***^ (0.117)	0.146^***^ (0.031)	0.145^***^ (0.031)
Chronic Diseases	−0.011 (0.046)	0.033 (0.046)	−0.007 (0.014)	0.015 (0.014)
Hospitalized	−0.081 (0.050)	−0.048 (0.051)	−0.014 (0.015)	0.005 (0.016)
Self-rated health				
Excellent		0.648^***^ (0.078)		0.331^***^ (0.023)
Very good		0.466^***^ (0.072)		0.217^***^ (0.022)
Good		0.310^***^ (0.063)		0.125^***^ (0.019)
Fair		0.171^**^ (0.071)		0.059^***^ (0.020)
Education dummy	Yes	Yes	Yes	Yes
Year dummy	Yes	Yes	Yes	Yes
Province dummy	Yes	Yes	Yes	Yes
_cons	7.918^***^ (1.799)	7.240^***^ (1.855)	3.993^***^ (0.434)	3.744^***^ (0.440)
*N*	46,919	46,916	77,486	77,482

HME, household medical expenses; HPE, household health protection expenses; HCE, household consumption expenses. Robust standard errors in parentheses. ^*^p < 0.1, ^**^p < 0.05, ^***^p < 0.01.

In models 3 and 4, the dependent variable was life satisfaction. The fixed-effects model that did not include self-rated health (model 3) showed that the impact of household medical expenses on life satisfaction was significant at the 10% significance level. However, the effect of household medical expenses on life satisfaction was not significant when self-rated health was included in the model (model 4). The health status of an individual is related to medical expenses, and this could affect the life satisfaction of an individual. The omission of health status can therefore cause omitted variable bias, leading to inconsistent estimation results. Therefore, the results of model 3 may be inconsistent, while those of model 4 are likely more reliable. The fixed-effects modeling revealed a negative effect of household health protection expenses on life satisfaction at the 10% significance level, but the effect was very small. In model 4, with other conditions remaining unchanged, the average life satisfaction of individuals with household health protection expenditure was 0.024 lower compared with those that did not have household health protection expenditure.

The results of fixed-effects modeling showed that the effect of household medical expenses on happiness and life satisfaction was not significant, while household health protection expenses had a significant negative impact on the happiness and life satisfaction of individuals. These results are inconsistent with the results of descriptive statistics. The results of descriptive statistics are a simple correlation analysis between two variables, which does not consider the impact of other variables. Compared with correlation analysis between two variables, fixed-effects models are more reliable and robust, helping to solve the omitted variable bias to a certain degree.

What are the potential reasons behind these findings? An increase in household health expenditure will reduce the real household income of residents. Real household income has been shown to have a significant positive impact on personal SWB in China (42–44), and the results presented herein also support this viewpoint. This real household income will be used for other consumption or savings to achieve utility and SWB, so reduced real household income will lower personal SWB; this is here termed a real income inhibitory effect. An increase in household health expenditure will improve the health status of individuals, and improved health status will enhance personal SWB; existing research and our findings herein both support this viewpoint (43, 45), and this is here termed a health promotion effect. The effect of household health expenditure on personal SWB is then a combination of the two aforementioned effects. According to the results presented herein, the impact of household medical expenses on the happiness and life satisfaction of individuals is not significant, with real income inhibitory and health promoting effects reaching a balance. Meanwhile, the impact of household health protection expenses on the happiness and life satisfaction of individuals is negative, with the real income inhibitory effect being greater than the health promoting effect.

The impact of household health consumption on personal SWB may be related to gender. Therefore, we evaluated the effect of household health consumption on personal SWB separately using female and male sub-samples, with the results shown in Table 5. The effects of household medical expenses on the happiness and life satisfaction of individuals were not significant in either sub-sample; this is consistent with the results obtained from the full sample. However, the effects of household health protection expenses on SWB were different for different genders. For the female sub-sample, individuals whose families had health protection expenses had lower average happiness and life satisfaction than those individuals whose families did not have health protection expenses. For the male sub-sample, the effects were not significant.

**Table 5:** Fixed effect model regression results of different gender.

	Female	Male	Female	Male
	Happiness	Happiness	Life satisfaction	Life satisfaction
Ln HME	−0.010 (0.008)	0.005 (0.008)	0.000 (0.002)	−0.004 (0.003)
HPE dummy	−0.170^***^ (0.056)	−0.062 (0.052)	−0.043^**^ (0.018)	−0.006 (0.017)
Ln household income	0.082^***^ (0.029)	0.078^**^ (0.037)	0.014 (0.009)	0.028^***^ (0.010)
Ln HCE	0.045 (0.038)	−0.011 (0.036)	0.009 (0.011)	−0.006 (0.011)
Self-rated health				
Excellent	0.657^***^ (0.108)	0.627^***^ (0.112)	0.298^***^ (0.032)	0.379^***^ (0.034)
Very good	0.485^***^ (0.100)	0.441^***^ (0.103)	0.161^***^ (0.029)	0.286^***^ (0.032)
Good	0.329^***^ (0.086)	0.279^***^ (0.091)	0.078^***^ (0.025)	0.186^***^ (0.028)
Fair	0.218^**^ (0.097)	0.108 (0.103)	0.040 (0.027)	0.090^***^ (0.030)
Age	0.035 (0.088)	−0.027 (0.047)	−0.023 (0.021)	−0.014 (0.010)
Urban	0.146 (0.109)	0.101 (0.118)	0.016 (0.035)	−0.023 (0.034)
Marriage dummy	0.243 (0.164)	0.430^***^ (0.166)	0.095^**^ (0.043)	0.198^***^ (0.044)
Chronic diseases	0.028 (0.065)	0.039 (0.066)	0.021 (0.020)	0.009 (0.020)
Hospitalized	−0.059 (0.072)	−0.032 (0.070)	0.001 (0.021)	0.010 (0.022)
Education dummy	Yes	Yes	Yes	Yes
Year dummy	Yes	Yes	Yes	Yes
Province dummy	Yes	Yes	Yes	Yes
_cons	5.544 (3.806)	6.737^***^ (2.146)	4.279^***^ (0.936)	3.724^***^ (0.492)
*N*	23,515	23,401	38,840	38,642

HME, household medical expenses; HPE, household health protection expenses; HCE, household consumption expenses. Robust standard errors in parentheses. ^*^p < 0.1, ^**^p < 0.05, ^***^p < 0.01.

Female led family wealth management rights in China (46). Married women in China have a stronger sense of family responsibility, which make them to reduce unnecessary expenses and improve the SWB of the entire family. The data in this article shows that the female that have a spouse in marriage account for 76.98% of all female samples. This might cause females to be more sensitive to household health protection expenses, which might cause a negative impact of household health protection expenses on their sense of SWB.

### 3.3 Robustness testing

Fixed effects IV regression was used to conduct robustness testing. Drawing on existing research ideas (47), the lagged natural logarithm of household medical expenditure was selected as the IV. The panel data used here included surveys from 2016, 2018, and 2020; hence, lagged by two orders was selected as the IV. Lagged household medical expenditure is related to current household medical expenditure. Owing to the lagged variable being predetermined (its value has been fixed from the current perspective), it may not correlate with the error term. Therefore, the lagged variable may be a suitable IV. The results are shown in Table 6, where the Cragg–Donald–Wald *F*-test shows that the selected IV is related to household medical expenses with strong significance; the IV is not weak identification. After considering potential endogeneity issues, the relationship between household medical expenses and personal SWB remained insignificant, this is completely consistent with the previous results. Household health protection expenses had a negative impact on happiness at the 5% significance level, while the impact of health protection expenses on life satisfaction was negative at the 10% significance level. These results are consistent with the individual fixed-effects model results shown in Table 4.

**Table 6:** Results of fixed effects instrumental variable (IV) regression and propensity-score matching (PSM).

	Model 1	Model 2	Model 3	Model 4
	Fixed effects IV	Fixed effects IV	PSM (ATT)	PSM (ATT)
Dependent variable	Happiness	Life satisfaction	Happiness	Life satisfaction
Ln HME	0.013 (0.014)	0.010 (0.007)		
HPE dummy	−0.111^**^ (0.045)	−0.026^*^ (0.015)	−0.080^**^ (0.035)	−0.003 (0.014)
Ln household income	0.077^***^ (0.023)	0.021^***^ (0.007)		
Ln HCE	0.006 (0.029)	−0.011 (0.010)		
Age	−0.016 (0.032)	−0.008 (0.010)		
Urban	0.112 (0.096)	0.003 (0.027)		
Female	0.127 (0.619)	0.185 (0.192)		
Marriage dummy	0.349^***^ (0.110)	0.146^***^ (0.031)		
Chronic diseases	0.020 (0.047)	0.017 (0.014)		
Hospitalized	−0.082 (0.052)	−0.004 (0.017)		
Self-rated health				
Excellent	0.656^***^ (0.074)	0.325^***^ (0.023)		
Very good	0.496^***^ (0.069)	0.220^***^ (0.021)		
Good	0.308^***^ (0.058)	0.117^***^ (0.018)		
Fair	0.190^***^ (0.065)	0.054^***^ (0.019)		
Education dummy	Yes	Yes		
Year dummy	Yes	Yes		
Province dummy	Yes	Yes		
_cons	7.183^***^ (1.547)	3.543^***^ (0.501)		
*N*	39,409	65,727		
Cragg–Donald Wald *F* statistic	2778.749	2966.112		
10% maximal IV size	16.38	16.38		

HME, household medical expenses; HPE, household health protection expenses; HCE, household consumption expenses; ATT, average treatment effect on the treated. Standard errors in parentheses. ^*^p < 0.1, ^**^p < 0.05, ^***^p < 0.01.

The PSM method was adopted to study the impact of household health protection expenses dummy on SWB to conduct further robustness testing. The standardized differences of all covariates after matching were less than 0.03, these being much lower than those before matching; hence, the matching results met the balance requirements well. The estimated PSM results are shown in Table 6, it can be seen that individuals whose families had health protection expenses had lower levels of happiness; household health protection expenses had a significant negative impact on the happiness of individuals. The impact of household health protection expenses on the life satisfaction of individuals was also negative, but the impact was very small and insignificant. The fixed-effect modeling revealed a negative effect of household health protection expenses on life satisfaction only at the 10% significance level, and the effect was very small (Table 4). Hence, the negative impact of household health protection expenses on the life satisfaction of individuals may not be robust.

## 4 Discussion

The effect of household health expenditure on personal SWB might a combination of two opposite effects. The two opposite effects are real income inhibitory effect and health promotion effect respectively, that we have explained in section 3.2 of this article. According to the results presented herein, the impact of household medical expenses on the happiness and life satisfaction of individuals is not significant, with real household income inhibitory and health promoting effects reaching a balance. Household medical expenses can reduce other family consumption and savings levels, they have a significant negative impact on residents’ well-being. However, most household medical expenditures are mandatory and have rigidity. Residents have a certain degree of psychological expectations for the health expenditures, that can reduce the degree of this negative impact. Adding household medical expenses can improve health conditions, causing household medical expenses on the happiness and life satisfaction of individuals are not significant. Meanwhile, the impact of household health protection expenses on the happiness and life satisfaction of individuals is negative. Many household health protection expenses are not necessary expenditures, improving health can be achieved through many free healthcare activities, which result in the real income inhibitory effect being greater than the health promoting effect.

For the female sub-sample, individuals whose families had health protection expenses had lower average happiness and life satisfaction than those individuals whose families did not have health protection expenses. For the male sub-sample, the effects were not significant. For this gender difference, we attempt to explain using the responsibility possessed by Chinese female. More than 80% of young women are in charge of household finances in China, that is far exceeding the global average level (48). In order to prevent potential funding issues and prevent problems before they arise, married women need to carefully and responsible for daily expenses. And females are more risk averse than males (49, 50), which make them to reduce unnecessary expenses to ensure the safety of family life. This might cause females to be more sensitive to household health protection expenses than males for many household health protection expenses are not necessary expenditures.

There are few researches about the impact of household health consumption on residents’ SWB. However, there are many studies about the effect of government health expenditure on residents’ SWB. Most studies suggest that government health expenditure could improve people’s SWB in other countries (24, 25, 51), there are similar conclusions in China (23, 52). Public health investment is to reduce household health expenses that is conducive to releasing residents’ consumption demand and improving residents’ SWB (52). This indirectly confirms that household health expenditures cannot improve residents’ SWB to some extent.

The results reported herein show that household medical expenses do not enhance the SWB of residents. However, improving in health status could enhance people’s SWB. Chinese health care system is aim to reduce the household medical expenses and improve the health level of residents in China, that could enhance the SWB of all citizens through increase other consumptions or savings level, then to promote economic development and realize common prosperity.

The health of residents has always been highly valued by the Chinese government (53). The healthcare has made significant progress in China; according to the 2022 Statistical Bulletin on China’s Health and Hygiene Industry, at the end of 2022, there were 9.75 million beds in medical and health institutions nationwide, an increase of 300,000 beds compared with the previous year. The number of beds in medical and health institutions per thousand of the population increased from 6.70 in 2021 to 6.92 in 2022. Many hospitals in China are public hospitals (54); these hospitals are concerned with public welfare, undertaking public services, and are non-profit-seeking health institutions (55). The highest reimbursement rate for basic medical insurance is 95%, and the issue of basic medical security for residents has been resolved to a certain extent (56–59). According to the 2022 Statistical Bulletin on China’s Health and Hygiene Industry, in 2022, government health expenditure was 2,392 billion yuan, which accounted for 28.2% of total health expenditure, while health expenditure by individuals reached 2,291 billion yuan, which accounted for 27.0% of total health expenditure. All of these increased government’s public health investment that could reduce household medical expenses to enhance residents’ SWB.

The results presented herein show that household health protection expenses could lower the SWB of residents. At present, the health consumption of Chinese people is mainly concentrated in medical treatment, while health protection consumption is insufficient. According to CFPS 2020, the average household medical expenditure is 10.6 times that of the average household health protection expenditure. In 2020, the *per capita* consumption of health protection products in China was U.S. $28, less than half of the average global level (12). An increase in expenditure on health protection products will reduce the SWB of residents, which is contrary to the concept of “Happiness China.” In addition, health protection products in China are plagued by false advertising of product effectiveness, which could result in a negative impact on the SWB of consumers.

## 5 Policy implications

Improving the health and happiness of residents are important current concerns of the Chinese government. Although medical expenditure does not appear to improve the SWB of residents, medical expenditure could improve the health status of human capital, which could affect economic development (60, 61). By adhering to and optimizing the current policy of reducing the burden of household medical expenses, improving the social security system for all citizens, the health level of residents could be ensured, then other types of consumption could be effectively encouraged. The increased other consumption could promote economic development and increase people’s income, then promote a virtuous cycle to the entire national economy. These may enhance the SWB of residents while promoting economic development, achieving common prosperity for all residents. In addition, the medical industry has the characteristic of information asymmetry, it cannot rely solely on the market in order to reduce the medical burden on residents. This provides theoretical reference for the implementation of relevant policies in China.

According to CFPS, in 2020, only 20% of households in China had health protection expenses, indicating that preventive health consumption is insufficient (12). The results presented herein show that household health protection expenses could lower the happiness of residents. National Fitness Program (2021–2025) has been released in 2021; one of the tasks of this program is to increase the public supply of fitness facilities for all residents. The program will supervise local governments to formulate a five-year action plan to fill gaps in fitness facility construction, and implement the national fitness facility filling project. Popularization of national fitness venues and equipment could ensure the basic fitness needs of residents, reducing the fitness expenses of residents and improving their SWB. The results of this study provide support for the implementation of similar programs and policies.

Health protection products in China face challenges including insufficient product maturity and a lack of industry standards, as well as false advertising of product effectiveness (12). Hence, advertising related to the effectiveness of health protection products should be standardized, guiding residents to consume rationally and improve their well-being. The development of the healthcare product industry may need to be regulated to ensure that healthcare products truly improve personal health and well-being.

## 6 Limitations

First, the influence mechanism of household health expenditure on people’s SWB that is needed to further investigate and explore in the future. We analyzed possible reasons for gender difference of household health protection consumption on personal SWB, this still needs further investigation and analysis through empirical researches in the future.

Second, although CFPS is a tracking investigation, there are many missing values for different variables in each survey. This may affect the robustness of statistical results in this article.

Finally, only one instrumental variable was used for fixed effects instrumental variable (IV) regression to robustness testing in this article. The suitability of instrumental variables could only be tested through empirical judgment, that cannot be statistically analyzed. Future research may find more and better instrumental variables, as the search for instrumental variables is very difficult and requires some inspiration.

## 7 Conclusion

This study used panel data to investigate the impacts of household medical and health protection expenditure on personal SWB. The results reported herein lead to the conclusion that household medical and health protection expenditure does not improve personal SWB; indeed, household health protection consumption could significantly reduce personal happiness. And household health protection consumption has a greater negative impact on female than male. These results have certain significance for the formulation of relevant policies.

## Data Availability

The data analyzed in this study is subject to the following licenses/restrictions: the raw data of China Family Panel Studies (CFPS) could free to download and conduct academic research, but the processed data is not allowed to be publicly published. Requests to access these datasets should be directed to http://www.isss.pku.edu.cn/cfps/gycfps/cfpsjj/index.htm.
